# Accuracy of the nursing diagnosis of ineffective airway clearance in intensive care unit patients

**DOI:** 10.1590/0034-7167-2022-0174

**Published:** 2023-01-30

**Authors:** Juliane Rangel Dantas, Anna Thays Dias Almeida, Karolayne Cabral Matias, Maria Isabel da Conceição Dias Fernandes, Jéssica Dantas de Sá Tinôco, Marcos Venícios de Oliveira Lopes, Ana Luísa Brandão de Carvalho Lira

**Affiliations:** I Universidade Federal do Rio Grande do Norte. Natal, Rio Grande do Norte, Brazil; II Universidade Estadual do Rio Grande do Norte. Caicó, Rio Grande do Norte, Brazil; III Universidade Federal do Ceará. Fortaleza, Ceará, Brazil

**Keywords:** Intensive Care Units, Respiratory System, Nursing Diagnosis, Data Management, Nursing., Unidades de Terapia Intensiva, Sistema Respiratório, Diagnóstico de Enfermagem, Gestão de Dados, Enfermagem., Unidades de Cuidados Intensivos, Sistema Respiratorio, Diagnóstico de Enfermería, Gestión de Datos, Enfermería.

## Abstract

**Objectives:**

to analyze the accuracy of the clinical indicators of ineffective airway clearance in adult intensive care unit patients.

**Methods:**

diagnostic accuracy study, performed in the intensive care unit of a university hospital in northeastern Brazil. The sample consisted of 104 patients hospitalized between June and October 2019.

**Results:**

the prevalence of ineffective airway clearance was 36.54%. The indicators with high specificity included absence of cough (0.8326), orthopnea (0.6817), adventitious breath sounds (0.8175), and diminished breath sounds (0.8326). The clinical indicators with high sensitivity and specificity were alteration in respiratory rate (0.9999) and alteration in respiratory pattern (0.9999).

**Conclusions:**

six clinical indicators provided an accurate identification of ineffective airway clearance. The clinical indicators alteration in respiratory rate and alteration in respiratory pattern were the most accurate for critical adult patients. The findings of this study contribute to accurate diagnostic inferences and to prevention of respiratory complications in these patients.

## RESUMO

**Objetivos:**

analisar a acurácia dos indicadores clínicos de desobstrução ineficaz de vias aéreas em pacientes de unidade de terapia intensiva adulto.

**Métodos:**

estudo de acurácia diagnóstica em unidade de terapia intensiva de um hospital universitário do nordeste do Brasil. Amostra de 104 pacientes internados entre junho e outubro de 2019.

**Resultados:**

a prevalência de desobstrução ineficaz de vias aéreas foi de 36,54%. Os indicadores com alta especificidade foram ausência de tosse (0,8326), ortopneia (0,6817), sons respiratórios adventícios (0,8175) e sons respiratórios diminuídos (0,8326). Os indicadores clínicos com alta sensibilidade e especificidade foram alteração na frequência respiratória (0,9999) e alteração no padrão respiratório (0,9999).

**Conclusões:**

seis indicadores clínicos forneceram identificação precisa da desobstrução ineficaz de vias aéreas. Os indicadores clínicos alteração na frequência respiratória e alteração no padrão respiratório foram os mais precisos para pacientes críticos. Os achados contribuem para inferências diagnósticas precisas e para prevenção de complicações respiratórias nesses pacientes.

## RESUMEN

**Objetivos:**

analizar la precisión de indicadores clínicos de limpieza ineficaz de las vías aéreas en pacientes de unidades de cuidados intensivos adulto.

**Métodos:**

estudio de precisión diagnóstica en unidad de cuidados intensivos de hospital universitario en noreste brasileño. Muestra de 104 pacientes hospitalizados entre junio y octubre de 2019.

**Resultados:**

prevalencia de limpieza ineficaz de las vías aéreas del 36,54%. Indicadores con alta especificidad; ausencia de tos (0,8326), ortopnea (0,6817), ruidos respiratorios adventicios (0,8175) y ruidos respiratorios disminuidos (0,8326). Indicadores clínicos con alta sensibilidad y especificidad; cambio en la frecuencia respiratoria (0,9999) y cambio en el patrón de respiración (0,9999).

**Conclusiones:**

seis indicadores clínicos proporcionaron identificación precisa de la limpieza ineficaz de las vías aéreas; cambio en la frecuencia respiratoria y cambio en el patrón de respiración fueron los más precisos para pacientes críticos. Nuestros hallazgos contribuyen a inferencias diagnósticas precisas y la prevención de complicaciones respiratorias en estos pacientes.

## INTRODUCTION

Technological advances have directly impacted the improvement and effectiveness of the care process^(1)^, especially in the treatment of critically ill patients. However, the rates of mortality and morbidity due to respiratory problems continue to increase worldwide^(2)^.

Respiratory problems are often diagnosed in intensive care unit patients. Studies showed that mechanical ventilation problems and respiratory dysfunctions were the main causes of prolonged hospital stay and mortality^(3-4)^.

In 2020, this reality became even more evident due to the COVID-19 pandemic^(5)^. COVID-19 patients can develop severe pneumonia and progress to respiratory failure with continuous ventilatory support in an intensive care unit^(6)^. Human responses such as impaired gas exchange, ineffective airway clearance, decreased cardiac output, and acute pain are frequently manifested by these patients^(7-8)^.

Ineffective airway clearance (IAC) is a major cause of reduced blood oxygenation and removal of circulating carbon dioxide. The patient with IAC may need intubation and sedation^(8-10)^. The nursing diagnosis of IAC is defined as the inability to clear secretions or obstructions from the respiratory tract to maintain a clear airway. Its main causes are infection and inflammation. The diagnosis has a level of evidence of 3.3 according to NANDA-I and was revised in 2020. Studies with higher levels of evidence are needed to improve the NANDA-I taxonomy^(11-12)^.

The studies identified the presence of IAC in critical adult patients^(13-15)^. Other studies carried out in Brazil^(16-17)^ identified IAC in pediatric patients with respiratory diseases. Despite identifying the prevalence of IAC in adults and its accuracy in children, the previous studies did not describe the accurate clinical indicators in adult ICU patients. Thus, conducting studies that seek these indicators in critically ill adult patients can fill this gap and make this reality clearer.

Thus, there is a need for further studies with robust evidence on IAC’s accuracy in adult ICU patients. These studies can help nurses by providing elements that are central for an accurate diagnostic reasoning and helping to minimize complications.

Ventilatory care for patients with IAC involves a sum of activities that will be defined based on the nurse’s assessment^(18)^. An accurate nursing assessment requires the identification of the clinical indicators that best represent the patients’ actual problems^(19)^. The identification of accurate clinical indicators assists nurses in their diagnostic inferences and effective nursing interventions.

## OBJECTIVES

To analyze the accuracy of the clinical indicators of ineffective airway clearance in adult intensive care unit patients.

## METHODS

### Ethical aspects

During the study, written permission was obtained from the Ethics Committee of the university in which the study was undertaken. Written consent was obtained from all participants or their legal guardians.

### Study design, period and place

This was a diagnostic accuracy study. The study was conducted and structured with reference to the “STARD 2015: An updated list of essential items for reports of diagnostic accuracy studies”^(20)^. The data collection was performed between June and October 2019 in a university hospital in northeastern Brazil.

### Population or sample, inclusion and exclusion criteria

This sample was calculated based on a study that proposed the use of a number of individuals for each clinical indicator. Thus, eight individuals were used for each of the thirteen clinical indicators of the IAC nursing diagnosis^(21)^.

In total, 104 adults admitted to the intensive care unit of a university hospital in northeastern Brazil participated in the study. Inclusion criteria were patients aged 18 years or older and admitted to the hospital’s intensive care unit. The exclusion criteria were unconscious patients without the guardian’s presence, and ICU readmission during the study.

### Study protocol

Data collection was performed by a research nurse. The techniques used were direct observation, anamnesis and directed physical examination. The evaluation sources were the patient, legal guardian and medical records. The instruments used were stethoscope, pulse oximeter and clipboard.

The 11th edition of NANDA-I taxonomy was used to construct the instrument and protocol for this research^(22)^. The data collection instrument was a questionnaire including sociodemographic (age, gender, marital status, years of study, occupation) and clinical data (use of artificial airway, respiratory data, data smoking) and variables concerning the indicator clinical indicators of IAC. The following clinical indicators were assessed: alteration in respiratory rate, alteration in respiratory pattern, absence of cough, ineffective cough, cyanosis, difficulty verbalizing, dyspnea, excessive sputum, restlessness, wide-eyed look, orthopnea, adventitious breathing sounds, and diminished breath sounds.

A protocol with the conceptual and operational definitions of the clinical indicators under investigation was used to guide the instrument’s construction. The instrument was subjected to a pre-test with 10% of the sample and showed no need for changes or improvements.

### Analysis of results and statistics

The data were organized in Microsoft Office Excel 2010 and analyzed using the R software version 2.12.1. The descriptive analyses were performed based on absolute and relative frequencies, central tendency and dispersion measures. The Kolmogorov-Smirnov test was used to analyze the normality of the data. A p-value <0.05 was considered statistically significant.

The accuracy of the clinical indicators was analyzed using a random-effect latent class model. The aims of this model are to identify unobservable (latent) variables and to determine an association with observable variables^(23)^. In this study, the latent variable was the diagnosis of IAC and the observable variables were the clinical indicators.

The accuracy measures identified were sensitivity and specificity. Sensitivity refers to the number of individuals with the diagnosis of interest who have a given clinical indicator. Specificity is defined as the number of individuals without the diagnosis of interest who do not have a given clinical indicator. The likelihood ratio test (G2) was used to identify the statistical significance between the clinical indicators^(19)^.

From the adjusted latent class model, subsequent probabilities of the occurrence of the nursing diagnosis were calculated for each different arrangement of the clinical indicators that compose this model. These calculations allow establishing the probability of the diagnosis being present/absent according to the presence/absence of different combinations of clinical indicators. The diagnosis was considered present when the later probability of this status was greater than 0.5.

## RESULTS

### Participant characteristics

In the study, a total of 104 patients were surveyed. The patients’ median age was 61 years; 54.8% were male, 59.6% were married, 72.1% were retired, with a median of eight years of study, 60.6% were smokers, and 54.8% reported secondhand smoke. Most patients (77.9%) were not using the airway at the time of data collection but 19,2% used an endotracheal tube and 2,9% used a tracheostomy.

The most frequent clinical indicators related to ineffective airway clearance were dyspnea (43.3%), ineffective cough (42.3%), alteration in respiratory rate (36.5%), alteration in respiratory pattern (36.5%), and orthopnea (33.7%) (Table 1).

**Table 1:** Frequency of clinical indicators of ineffective airway clearance in patients in an intensive care unit

Clinical indicators	n	%	95%CI
Dyspnea	45	43.3	33.7-53.3
Ineffective cough	44	42.3	32.8-52.3
Alteration in respiratory rate	38	36.5	27.4-46.6
Alteration in respiratory pattern	38	36.5	27.4-46.6
Orthopnoea	35	33.7	24.8-43.6
Difficulty verbalizing	26	25.0	17.2-34.6
Adventitious breath sounds	24	23.1	15.6-32.5
Absence of cough	21	20.2	13.2-29.4
Diminished breath sounds	16	15.4	9.3-24.0
Restlessness	10	9.6	4.96-17.3
Excessive sputum	06	5.8	2.36-12.6
Wide-eyed look	03	2.9	0.74-8.81
95% CI: 95% confidence interval.			

### Diagnostic accuracy measures

The clinical indicators alteration in respiratory rate and alteration in respiratory pattern had high sensitivity values. On the other hand, alteration in respiratory rate, alteration in respiratory pattern, absence of cough, orthopnea, adventitious breath sounds, and diminished breath sounds had high specificity values (Table 2).

**Table 2:** Accuracy measures of the clinical indicators of ineffective airway clearance in patients in an intensive care unit

Clinical indicators	Se	95%CI		Sp	95%CI	
Alteration in respiratory rate	0.9999	0.9999	1.0000	1.0000	0.9999	1.0000
Alteration in respiratory pattern	0.9999	0.9999	1.0000	1.0000	0.9999	1.0000
Absence of cough	0.2617	0.1427	0.4672	0.8326	0.6844	0.9057
Orthopnoea	0.3674	0.2441	0.5332	0.6817	0.5501	0.7779
Adventitious breath sounds	0.3145	0.193	0.4808	0.8175	0.6845	0.8872
Diminished breath sounds	0.1304	0.0278	0.532	0.8326	0.7155	0.9035
Prevalence of IAC: **36.54%**	G^2^: 54.93		DF: 50		p-value: 0.293	

*Se - sensitivity; Sp - specificity; CI - confidence interval; G2 - likelihood ratio test; DF - degrees of freedom.*

Clinical indicators with values close to 1 are considered more sensitive or specific. These will also have greater diagnostic accuracy. Thus, the most clinical indicators accurate of ineffective airway clearance are alteration in respiratory rate and alteration in respiratory pattern. The prevalence of the diagnosis of IAC was 36.54% (Table 2).

## DISCUSSION

The prevalence of IAC found in the present study was similar to that reported in the literature^(7-8,24)^. In the presence of the IAC diagnosis, the nurse must act in order to remove or minimize obstructions to facilitate the passage of air and promote gas exchange^(25)^.

Nursing diagnoses provide the basis for the planning and implementation of nursing interventions to achieve desired outcomes for patients^(26)^.The main interventions for patients with IAC include (1) to facilitate the secretion remotion by means of hydration, (2) patient mobilization, (3) stimulation to the cough, (4) secretion aspiration, and (5) control/monitorization of the respiratory condition^(25)^.

In the present study, the most accurate clinical indicators were alteration in respiratory rate and alteration in respiratory pattern. Compensatory respiratory mechanisms are activated in the presence of airway obstruction. The decrease in blood oxygenation activates the central nervous system, stimulating the respiratory muscles to increase the respiratory depth and rate^(27)^.

The clinical indicators mentioned above also had good accuracy values in studies developed with asthmatic children^(28)^ and children with acute respiratory failure^(17)^. In the adult clientele, these clinical indicators were found and reported in a study with patients in the cardiac postoperative period^(29)^; however, unlike the present study, they were not accurate.

The clinical indicator absence of cough was also relevant for the presence of IAC. The literature states that the cough reflex is responsible for the process of clearing the airways^(16,30)^. Study shows that the clinical indicators ineffective cough and absent cough had high specificity values in patients in the cardiac postoperative period and their presence was related to immobility, sedative effect, pain, and history of smoking^(29)^.

The absence of cough in critically ill adult patients reduces the sputum and increases the occurrence of IAC. Study shows that the nursing prescription for stimulating cough and sputum is appropriate for these patients^(25)^. However, another study^(31)^ revealed that these interventions are performed predominantly by physiotherapists.

In the present study, the clinical indicator orthopnea was specific, different from the findings of a survey conducted with asthmatic children, in which orthopnea had a high sensitivity value increasing the chances of having IAC by five^(28)^.

Orthopnea is the difficulty in breathing in the supine position, which can be alleviated by raising the patient’s bed head. This action allows greater pulmonary expandability by decreasing the load generated by the chest wall’s weight^(16)^.

The indicators adventitious breath sounds and diminished breath sounds occur due to the presence of secretions or fluids in the airways, leading to changes in the sounds produced in the respiratory tract^(17,28)^.

In previous studies^(29,32)^, adventitious breath sounds had a high sensitivity value, different from what has been found in the present investigation. Thus, adventitious breath sounds were the most accurate clinical indicators^(29)^. Similar to our findings, in others studies the clinical indicator diminished breath sounds had a high specificity^(28)^ and a high prevalence^(29)^ for IAC.

The accuracy measures found in the present study did not always present similar results in the literature. This can be explained by the fact that these studies are carried out in specific contexts, which reflects the need for further research in similar settings, such as the intensive care unit.

### Study limitations

In this study, the data were collected cross-sectionally and in a specific population of patients from a general ICU in Brazil. Thus, the results of this study cannot be generalized to all ICUs. The other limitation is related to the existence of few studies about the diagnosis of IAC in adult ICU patients, which limits the comparison of the results found in the present study.

### Contributions to Nursing, Health, or Public Policy

Although there are already routine interventions in the ICU for patients with IAC, it is important for nurses to reliably identify the signs and symptoms to infer a correct diagnosis. The findings of this study contributes to improve diagnostic inferences.

Accurate diagnostic inferences streamline the decision-making and lead to an improved prognosis for patients. This improves the clinical management of respiratory complications, streamlines activities with greater safety and allows for a reduction in hospitalization stay. Thus, the health system can be benefited with more efficient nursing care and a greater chance of promoting the health and well-being of hospitalized individuals.

Studies of this nature contribute to updating the nursing taxonomies and for the advance of nursing science.

## CONCLUSIONS

The present study showed that six clinical indicators are related to an improved ability to correctly identify the presence of IAC in adult ICU patients. This diagnosis is more likely to occur among patients with alteration in respiratory rate and alteration in respiratory pattern. The results of the study revealed the need for investigations about IAC in different contexts.
